# Emerging Disparities in Dietary Sodium Intake from Snacking in the US Population

**DOI:** 10.3390/nu9060610

**Published:** 2017-06-17

**Authors:** Elizabeth K. Dunford, Jennifer M. Poti, Barry M. Popkin

**Affiliations:** 1 Food Policy Division, The George Institute for Global Health, University of New South Wales, Sydney, NSW 2042, Australia; 2 Carolina Population Center, The University of North Carolina at Chapel Hill, Chapel Hill, NC 27516, USA; poti@unc.edu (J.M.P.); popkin@unc.edu (B.M.P.); 3 Department of Nutrition, The University of North Carolina at Chapel Hill, Chapel Hill, NC 27516, USA

**Keywords:** sodium intake, snacking, race-ethnic disparities

## Abstract

Background: The US population consumes dietary sodium well in excess of recommended levels. It is unknown how the contribution of snack foods to sodium intake has changed over time, and whether disparities exist within specific subgroups of the US population. Objective: To examine short and long term trends in the contribution of snack food sources to dietary sodium intake for US adults and children over a 37-year period from 1977 to 2014. Methods: We used data collected from eight nationally representative surveys of food intake in 50,052 US children aged 2–18 years, and 73,179 adults aged 19+ years between 1977 and 2014. Overall, patterns of snack food consumption, trends in sodium intake from snack food sources and trends in food and beverage sources of sodium from snack foods across race-ethnic, age, gender, body mass index, household education and income groups were examined. Results: In all socio-demographic subgroups there was a significant increase in both per capita sodium intake, and the proportion of sodium intake derived from snacks from 1977–1978 to 2011–2014 (*p* < 0.01). Those with the lowest household education, Non-Hispanic Black race-ethnicity, and the lowest income had the largest increase in sodium intake from snacks. While in 1977–1978 Non-Hispanic Blacks had a lower sodium intake from snacks compared to Non-Hispanic Whites (*p* < 0.01), in 2011–2014 they had a significantly higher intake. **Conclusions:** Important disparities are emerging in dietary sodium intake from snack sources in Non-Hispanic Blacks. Our findings have implications for future policy interventions targeting specific US population subgroups.

## 1. Introduction

Strong evidence links excessive dietary sodium intake to elevated blood pressure, which is a major risk factor for cardiovascular disease in both children and adults alike [1,2,3,4]. Research has also shown that in the US there are large and persistent disparities among race/ethnic subgroups related to hypertension, with the prevalence remaining highest among Non-Hispanic Black adults [5]. Despite the overall improvement of blood pressure control over the past 10 years, Non-Hispanic Blacks and Hispanics continue to have lower control rates than Non-Hispanic Whites [5].

The American Heart Association (AHA), World Health Organization (WHO), and Institute of Medicine all support recommendations to reduce population intake of sodium in the US [4,6,7]. Nonetheless, mean sodium intake in both US adults and children remains too high [8], with approximately two thirds of dietary sodium intake derived from packaged food sources [9,10]. We have shown in a recent publication, that although the sodium content of households’ total packaged food purchases decreased significantly between 2000 and 2014, mean sodium intake from packaged food purchases remained far above the AHA recommended intake levels, and the sodium content of processed foods exceeded the 2014 targets established by the National Salt Reduction Initiative [11]. 

Obesity increases over the past few decades have been attributed to a number of factors, one of which is to an increasing trend of snacking. Coupling this with the fact that the US population is consuming sodium levels in excess of dietary guidelines, it is likely that these increases in snacking behavior are contributing to both excessive dietary sodium intake overall in the US population, as well as within race-ethnic and socio-economic groups, in which health inequality has risen in recent decades [12]. Studies to date have observed inconsistent results in intakes of snacks between different race-ethnic groups and age and weight groups [13], and also demonstrate that a large proportion of snacks still appear to derive from less healthy foods, such as salty snacks, desserts and sweets [14,15]. One study even found that a single snack food item contributed, on average, 14% of a child’s daily recommended amount of sodium, with some items providing more than 150% [16] despite data from the most recent National Health and Nutrition Examination Survey (NHANES) indicating that on average, snack food sources contribute 16% of daily sodium intake in children six years and older [17].

To date, there have been no studies examining long term and short term trends in the amount and proportion of daily sodium intake derived from snack food sources, by demographic subgroups. Therefore, we have chosen to examine trends over a period of 37 years, for dietary sodium intake derived from snack food sources in US adults and children by age group, body mass index (BMI), race-ethnicity, household education and income level. 

## 2. Materials and Methods

### 2.1. Survey Population

Data were obtained from eight nationally representative surveys of food intake in 50,052 US children aged <19 years and 73,179 US adults aged 19+ years. The United States Department of Agriculture (USDA) data come from the 1977–1978 Nationwide Food Consumption Survey (NFCS 1977–1978), the 1989–1991 Continuing Survey of Food Intake by Individuals (CSFII 1989–1991), the 1994–1996 CSFII and the 1997–1998 CSFII (CSFII 1994–1998). From the NHANES, four surveys were used: NHANES 2003–2004, NHANES 2005–2006 (NHANES 03–06), NHANES 2011–2012 and NHANES 2013–2014 (NHANES 11–14). Supplementary Table S1 shows the number of records used in each survey year. The USDA and NHANES surveys are based on a multistage, stratified area probability sample of non-institutionalized US households. Detailed information about each survey and its sampling design has been published previously [18]. By utilizing secondary USDA and NHANES data, we were exempt from institutional review board concerns for this paper.

### 2.2. Snacking Definition

Each eating occasion was self-defined by the respondent in each survey. Respondents were asked to name the type of each eating occasion, and the main meal planner was asked about intake for any child under the age of 12 [19]. The snack category includes those eating occasions defined by the respondent as “snack” plus the occasions related to snacking, such as food and/or coffee/beverage breaks.

### 2.3. Dietary Data

All dietary survey data used a comparable food composition table and collection methods developed by the USDA. To examine trends over time from surveys with different collection methods on days 1 and 2, we used only the first day’s data (a single, 24-h dietary recall on the basis of interviews) collected from each individual, and used appropriate weights and adjustments for the sample design provided.

### 2.4. Food Grouping System

To determine those food items contributing to sodium intake from snacking, the food grouping system developed by the University of North Carolina at Chapel Hill (UNC-CH) was used. This food grouping system links all foods from 1977 to 2014. All the foods reported in the USDA surveys were assigned to the 107 UNC-CH food groups. The UNC-CH food grouping system has been described previously [20]. For all participants, the amount of sodium provided by each UNC-CH food group reported consumed as a snack was calculated and then divided by the total sodium from snacking of all individuals. Those food groups contributing the most to sodium intake from snack food sources are reported overall by year and by socio-demographic subgroup. 

### 2.5. Statistical Analysis

Data are presented as means (SE). Snacking trends were studied by dividing the population into age groups (2–5 years old, 6–11 years old, and 12–18 years old for children; 19–29 years old, 30–59 years old and 60+ years old for adults), BMI categories (Underweight, Normal Weight, Overweight and Obese, based on the WHO BMI guidelines), race-ethnic groups (Hispanic, Non-Hispanic White and Non-Hispanic Black), income groups using the Federal Poverty Level (FPL; <185% FPL, 185–350% FPL and >350% FPL) and household education groups (Less than High School, High School Diploma and More than High School). Income definitions used self-reported family income to compute the federal poverty level index. We calculated the mean sodium intake (mg) from snacks per capita per day, and the proportion of dietary sodium intake derived from snack foods overall for adults and children, and separately for each demographic subgroup. Stata version 14.1 was used for all analyses. Survey methods were used within Stata to account for the clustering and weighting that is inherent in the NHANES sampling methodology [21], so as to allow for statistically significant differences between survey cycles to be identified using Student’s *t*-test. A *p*-value of <0.05 was considered significant. 

## 3. Results

### 3.1. Overall Trends

For all US adults and children there was a significant increase in per capita sodium intake coming from snacks from 1977–1978 to 2011–2014 (*p* < 0.01) (Table 1). In all age groups the trend was an increase in sodium intake from snacks from 1977–1978 to 2003–2006, followed by a decrease from 2003–2006 to 2011–2014 (significant only in children; *p* < 0.01), except for those in the 60+-year-age group, which was the only group to show an increase in intake from 2003–2006 to 2011–2014 (Table 1). Despite a decrease observed for most age groups in the mean sodium intake derived from snacks from 2003–2006 to 2011–2014, there was little to no change seen in the proportion of the population that consumed snacks from 2003–2006 to 2011–2014.

### 3.2. Trends by Race-Ethnicity

**Figure 1:**
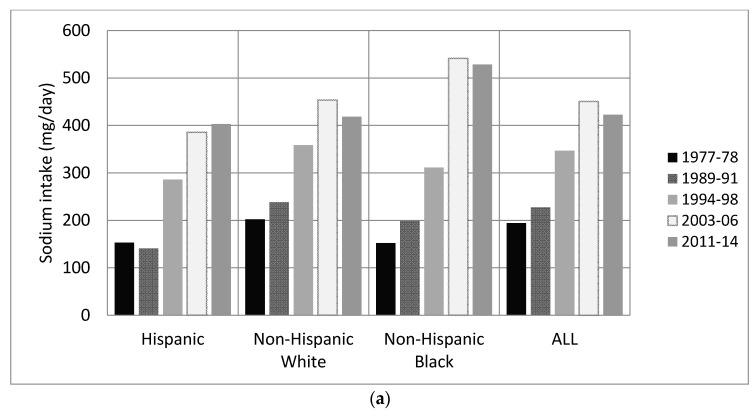

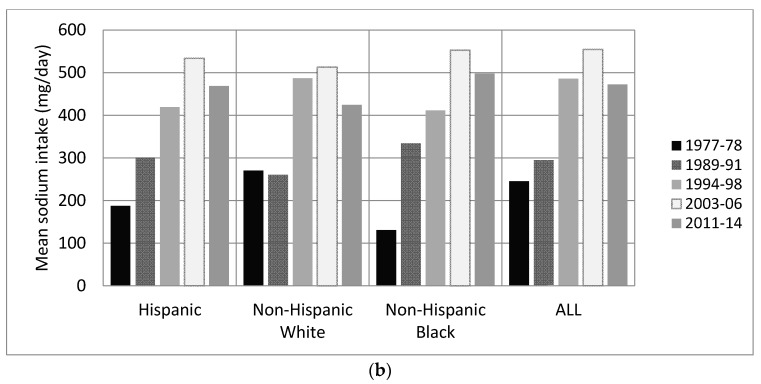
Sodium intake from snack food sources overall by race-ethnic group in US adults and children (**a**) Adults; (**b**) Children.

In all race-ethnic groups for both adults and children, there was an increase in sodium per capita per day from snack food sources between 1977–1978 and 2011–2014 (*p* < 0.01, Table 2), and a decrease from 2003–2006 to 2011–2014, although these decline results were only significant for Non-Hispanic White adults. While in 1977–1978 Non-Hispanic Black adults and children had a significantly lower sodium intake from snacks compared to Non-Hispanic Whites (*p* < 0.01 Figure 1a,b), in 2011–2014 Non-Hispanic Blacks had a significantly higher intake (*p* < 0.01) and had the highest mean sodium intake from snacks out of all race-ethnic groups from 2003 to 2006 onwards. Non-Hispanic Blacks also had the largest increase in mean sodium intake from snacks from 1977 to 2014, with the mean sodium intake more than doubling for Non-Hispanic Black adults and children over the study period.

### 3.3. Trends by Income Level and Education

**Figure 2:**
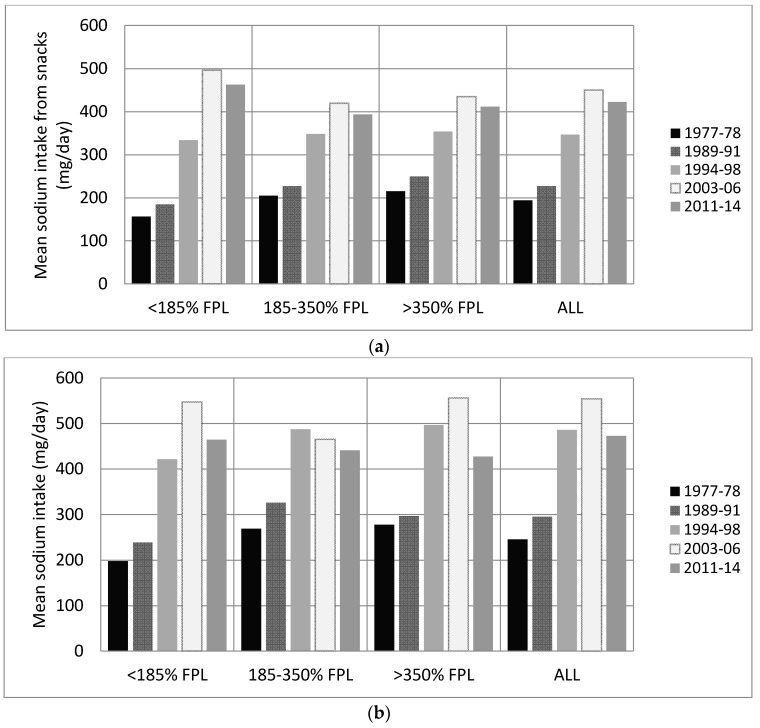
Sodium intake from snack food sources overall by income group in US adults and children (**a**) Adults; (**b**) Children. FPL, Federal Poverty Level.

All income and education groups showed an increase in sodium intake per capita per day from snacks between 1977–1978 and 2011–2014 (Table 2). The largest increase was seen in the lowest income group (<185% FPL) and the lowest education group (Less than high school). Adults and children in the lowest income group went from the lowest sodium intake from snacks to the highest from 1977 to 2014. Adults in the lowest income group went from a significantly lower intake of sodium from snacks than adults in the highest income group, to a significantly higher intake (*p* < 0.01; Figure 2a). Children in the lowest income group went from a significantly lower intake of sodium from snacks than children in the highest poverty level, to having no difference (Figure 2b). Those in the lowest household education group had the largest increase in sodium intake from salty snacks from 1977 to 2014 (Table 2). No important differences were observed when results were examined by BMI. Results can be seen in Supplementary Table S2.

### 3.4. Food Sources of Sodium Intake from Snacks

Salty snacks, and desserts and sweets, were the top two food group contributors to sodium intake from snacks in most survey years within most demographic subgroups (Supplementary Tables S3 and S4). Interestingly, either grain-based desserts or salty snacks were the number one contributor to sodium intake from snacks in each survey year in each race-ethnic group. Non-Hispanic Black adults and children had the largest increase in mean sodium intake from salty snacks from 1977 to 2014, compared to Hispanics and Non-Hispanic Whites (Supplementary Figures S1 and S2). 

## 4. Discussion

To the knowledge of the authors, this is the first study to examine both recent and long term trends in sodium intake derived from snack food sources in US adults and children in specific age, race-ethnic, household education and income groups. We found that in all socio-demographic subgroups there was a significant increase in both the per capita sodium intake, and the proportion of sodium intake derived from snacks from 1977–1978 to 2011–2014 (*p* < 0.01); however, in the most recent period following 2003–2006 we found a systematic decline in all socio-demographic subgroups. Most pronounced, were the trends in the Non-Hispanic Black race-ethnic group, that went from the lowest consumption of sodium from snacks to the highest intake of sodium from snacks, which is a marked shift in intake that adds to the evidence base indicating that disparities in sodium intake of this race-ethnic subpopulation are emerging. This was also seen in both adults and children in the lowest household education group of all ages, in sodium intake from snacks over the 37-year period.

A large proportion of sodium intake from snack foods derived from foods generally considered unhealthy, such as desserts/sweets and salty snacks. These two sources alone contributed to more than 25% of sodium from snack foods in both adults and children in 2014. This aligns with both a recent short term study in US children from 2003 to 2010, which showed that although a decrease in total calorie intake from discretionary foods had declined in recent years, that intake was still unacceptably high [13], and a recently published study examining long term trends in energy intake from snack food sources, which showed that grain-based desserts and salty snacks contributed greatly to daily energy intake in US children [22]. In fact, our data showed that for all years examined, either grain-based desserts or salty snacks were the number one source of sodium intake from snacking (Supplementary Table S3). Our findings are also supported by research from other countries which has shown, not only that the foods consumed as snacks are generally from less healthy food groups [23], but that disparities exist between various race-ethnic and education groups [24,25,26,27]. 

This study’s results showing that Non-Hispanic Blacks are consuming a higher intake of sodium from snacks than other race-ethnic groups is consistent with recent US research looking at behavioral shifts in food purchases, which found that Non-Hispanic Blacks were the only racial-ethnic group not to follow the overall trend of a decrease in per capita energy intake in the last decade [28] as well as research showing that Non-Hispanic Blacks, overall, have less healthy food purchasing behavior, in comparison to White and Hispanic populations [29]. However, another study examining food purchasing behavior found that Non-Hispanic Blacks actually purchased less sodium from store-bought packaged food sources than other race-ethnic groups, but also purchased much higher levels of salt-laden condiments and salt [30]. This difference might suggest that even though Non-Hispanic Black households may purchase less packaged food sources overall, they may be adding more salt during cooking and at the table [30], or are consuming snack foods from sources other than grocery stores.

Salty snack intake more than doubled from 1977–1978 to 2011–2014 overall in all US adults, and increased by ~75% in children (*p* < 0.01). However, we observed that although Non-Hispanic Blacks had the lowest overall sodium intake from salty snack food sources in 1977–1978, they had the highest sodium intake from salty snacks of all race-ethnic groups in 2011–2014. This is in contrast to prior research which found that the intake of salty snacks over the past 10 years has remained stable in children and adolescents [13], and another study which found that Non-Hispanic Whites had the highest intake of salty snacks [31]. However, prior studies in smaller samples of the US population have supported our results, showing less healthy snacking behaviors in Non-Hispanic Black adolescents [32,33] than in other groups. This, along with the high intake of sodium from salty snacks and desserts and sweets observed in Non-Hispanic Blacks in this study is concerning, with chronic disease risk factors shown to be considerably higher among Non-Hispanic Blacks compared to Non-Hispanic Whites [34]. 

Our analysis had some limitations. Collected dietary data has limitations in underreporting, particularly of food and beverages perceived as being less healthy, and these limitations can vary by age, race-ethnicity and body weight status [35,36,37]. Similar to other studies looking at US trends in dietary intake, different methodologies were used across different survey years. The introduction of the multiple pass method in the 1990s may have resulted in additional snacks identified during that period; however, our finding of a recent decrease in snacking gives us faith that this did not affect our overall findings. Earlier survey years conducted without the multiple pass method may be more prone to issues of recall bias. There is no bridging survey to help understand the impact of this methodological change, meaning that these surveys are the only ones available that use consistent food composition tables developed by the USDA specifically for the food supply at the time of each survey. Dietary intake assessment methods and food composition information have significantly improved over time. Although data presented are based on the first day 24-h recall from all surveys, the recall methodology was modified to include multiple passes through the list of foods and beverages in the CSFII 1994–1998 and to include the USDA’s automated collection system in NHANES surveys from 2002 onwards. Validation studies in adults have shown that these newer methods improved completeness of the recall [38]. As such, it is possible that the observed increases in sodium intake from 1977–1978 to 2003–2006 are an artefact of the more complete capture of the data. Each survey was linked to USDA food composition tables, but there may have been changes in nutrient composition based on different assay techniques, for which we cannot account [39,40]. These concerns were addressed by using the food grouping system developed by UNC-CH, which allows foods in each survey year to be linked to one consistent food group to offset changes in food composition table numbering, and to ensure high quality estimates of nutrient values over time [41].

## 5. Conclusions

Our study found a long term increase in overall sodium intake from snacking, but a declining trend in the last decade, to a lower intake in 2011–2014, and showed important disparities amongst Non-Hispanic Blacks, in particular, and differences between the lower household education and income groups. Importantly, we found that the major foods consumed at these snacking events (desserts and sweets, and salty snacks) are the foods recommended for reduced intake by the US dietary guidelines [42]. These only emphasized, again, the importance of not only focusing on reducing calories, but also improving diet quality. Our findings have major implications for future policy interventions targeting specific demographic subgroups of the US population.

## Figures and Tables

**Table 1:** Number of snacks consumed per day, percentage of snackers in the population, and sodium consumed per snacking occasion, by US children and adults from the 1977–1978, 1989–1991, 1994–1998, 2003–2006 and 2011–2014 surveys by age group.

	1977–1978	1989–1991	1994–1998	2003–2006	2011–2014
**Age 2–5 years**					
Snacks, *n*/day	1.2 (0.04) ^1^	1.4 (0.08) ^1^	2.3 (0.05) ^1^	3.0 (0.08)	3.0 (0.08)
Per capita mean intake from snacks, mg	157 (7.3) ^1^	211 (13.7) ^1^	389 (12.2) ^2^	444 (18.8)	443 (21.4)
% snackers	63 (1.7) ^1^	70 (2.9) ^1^	87 (1.2)	96 (0.6)	96 (0.6)
Per capita mean intake from snacks, mg (snackers only)	249 (8.8) ^1^	301 (17.1) ^1^	445 (11.4)	461 (18.2)	460 (21.8)
**Age 6–11 years**					
Snacks, *n*/day	1.0 (0.03) ^1^	1.1 (0.07) ^1^	1.8 (0.05) ^1^	2.7 (0.07)	2.5 (0.06)
Per capita mean intake from snacks, mg	206 (8.9) ^1^	281 (28.1) ^1^	454 (18.5)	562 (26.6) ^1^	468 (18.8)
% snackers	61 (1.3) ^1^	64 (2.4) ^1^	82 (1.3) ^1^	94 (0.9)	94 (0.9)
Per capita mean intake from snacks, mg (snackers only)	336 (12.9) ^1^	441 (37.7)	554 (18.5) ^2^	598 (29)	498 (20)
**Age 12–18 years**					
Snacks, *n*/day	1.0 (0.03) ^1^	1.1 (0.05) ^1^	1.7 (0.06)	2.3 (0.05)	2.1 (0.06)
Per capita mean intake from snacks, mg	314 (12.3) ^1^	367 (35.1) ^1^	575 (41)	606 (28.7) ^1^	492 (21.2)
% snackers	59 (1.4) ^1^	60 (2.2) ^1^	77 (1.7) ^1^	89 (0.9)	88 (1.1)
Per capita mean intake from snacks, mg (snackers only)	530 (17)	609 (53.2)	748 (40.8) ^1^	678 (29.8)	561 (24.8)
**Age 2–18 years**					
Snacks, *n*/day	1.1 (0.03) ^1^	1.2 (0.05)	1.9 (0.04)	2.6 (0.05)	2.4 (0.05)
Per capita mean intake from snacks, mg	245 (8.4) ^1^	295 (18.5) ^1^	486 (20.4)	554 (14.5) ^1^	472 (13.8)
% snackers	61 (1.3) ^1^	64 (1.9) ^1^	81 (1.2) ^1^	93 (0.5)	92 (0.6)
Per capita mean intake from snacks, mg (snackers only)	405 (11.4) ^1^	460 (23.2)	598 (19.5) ^1^	599 (14.9) ^1^	514 (15.6)
**Age 19–29 years**					
Snacks, *n*/day	1.1 (0.03) ^1^	1.1 (0.05) ^1^	1.6 (0.04) ^1^	2.2 (0.07)	2.2 (0.07)
Per capita mean intake from snacks, mg	251 (11.4) ^1^	273 (23.5) ^1^	473 (25.5)	575 (29.1) ^2^	486 (22.3)
% snackers	59 (1.3) ^1^	61 (1.8) ^1^	72 (1.2) ^1^	87 (1.3)	87 (1)
Per capita mean intake from snacks, mg (snackers only)	424 (15.4) ^1^	446 (32.7)	653 (35.5)	657 (35.1)	557 (25.6)
**Age 30–59 years**					
Snacks, *n*/day	1.2 (0.04) ^1^	1.3 (0.06) ^1^	1.6 (0.04) ^1^	2.4 (0.05)	2.5 (0.05)
Per capita mean intake from snacks, mg	196 (7.3) ^1^	238 (10.3) ^1^	345 (12.2) ^1^	466 (17.8)	440 (15.3)
% snackers	60 (1.3) ^1^	64 (1.6) ^1^	73 (1) ^1^	89 (0.6)	89 (0.8)
Per capita mean intake from snacks, mg (snackers only)	326 (9.3) ^1^	373 (13.5)	472 (12.8)	523 (18.4)	494 (15)
**Age 60+ years**					
Snacks, *n*/day	0.8 (0.02) ^1^	1.1 (0.05) ^1^	1.3 (0.04) ^1^	2.1 (0.05)	2.3 (0.07)
Per capita mean intake from snacks, mg	122 (5.9) ^1^	156 (11.3) ^1^	226 (9.2) ^1^	306 (11.7)	335 (16.4)
% snackers	49 (1.2) ^1^	58 (1.9) ^1^	68 (1.2) ^1^	88 (0.7)	88 (0.9)
Per capita mean intake from snacks, mg (snackers only)	251 (9.7) ^1^	270 (15.4)	332 (10.6)	348 (11.9)	381 (17.7)
**Age 19+ years**					
Snacks, *n*/day	1.0 (0.03) ^1^	1.2 (0.05) ^1^	1.5 (0.04) ^1^	2.3 (0.04)	2.4 (0.05)
Per capita mean intake from snacks, mg	194 (5.9) ^1^	227 (9.3) ^1^	347 (10.4) ^1^	450 (14.6)	423 (12.1)
% snackers	57 (1.1) ^1^	62 (1.4) ^1^	72 (0.9) ^1^	89 (0.6)	88 (0.6)
Per capita mean intake from snacks, mg (snackers only)	339 (7) ^1^	368 (10.9)	482 (11.5)	509 (15.5)	478 (12.7)

^1^ Different from 2011–2014, *p* < 0.01; ^2^ Different from 2011–2014, *p* < 0.05. Note: Data from the 1977–1978 Nationwide Food Consumption Survey (NFCS); the 1989–1991 Continuing Survey of Food Intake by Individuals (CSFII), the 1994–1996 CSFII, the 1997–1998 CSFII, NHANES 2003–2004, NHANES 2005–2006, NHANES 2009–2010 and NHANES 2011–2014. Results have been weighted to be nationally representative. Results are presented as mean (SE). NHANES, National Health and Nutrition Examination Survey.

**Table 2:** Number of snacks consumed per day, percentage of snackers in the population, and sodium consumed per snacking occasion, by US children and adults from the 1977–1978, 1989–1991, 1994–1998, 2003–2006 and 2011–2014 surveys by Federal Poverty Level, race-ethnicity and household education.

	Children					Adults				
	1977–1978	1989–1991	1994–1998	2003–2006	2011–2014	1977–1978	1989–1991	1994–1998	2003–2006	2011–2014
**<185% Federal Poverty Level**										
Snacks, *n*/day	0.9 (0.04) ^1^	1.2 (0.06) ^1^	1.9 (0.05)	2.5 (0.07)	2.3 (0.05)	0.8 (0.03) ^1^	1 (0.04) ^1^	1.3 (0.04) ^1^	2.1 (0.05)	2.2 (0.05)
Per capita mean intake from snacks, mg	198 (11.1) ^1^	239 (16.8) ^1^	421 (23.2)	547 (24.1) ^1^	464 (15.9)	156 (8.2) ^1^	185 (11.5) ^1^	334 (19.3) ^1^	496 (30)	463 (19.9)
% snackers	53 (1.9) ^1^	59 (2.1) ^1^	79 (1.5) ^1^	90 (0.7)	89 (0.7)	49 (1.5) ^1^	55 (1.7) ^1^	66 (1.2) ^1^	87 (0.7)	86 (0.8)
Per capita mean intake from snacks, mg (snackers only)	375 (18) ^1^	404 (19.6) ^1^	535 (27.2)	608 (24.9) ^1^	523 (16.2)	322 (12.7) ^1^	333 (14.5) ^1^	509 (26.6)	568 (33.6)	539 (22.6)
**185–350% Federal Poverty Level**										
Snacks, *n*/day	1.2 (0.03) ^1^	1.4 (0.09) ^1^	2.2 (0.06)	2.5 (0.09)	2.5 (0.08)	1.1 (0.03)	1.2 (0.07) ^1^	1.5 (0.04) ^1^	2.2 (0.06)	2.3 (0.06)
Per capita mean intake from snacks, mg	269 (11.2) ^1^	327 (30.4) ^1^	487 (35)	465 (23.7)	441 (25.7)	205 (6.8) ^1^	227 (15.3) ^1^	348 (17.6) ^2^	419 (19.2)	394 (21.9)
% snackers	65 (1.2) ^1^	69 (3.4) ^1^	83 (1.4) ^1^	90 (1)	88 (1.9)	60 (1.1) ^1^	59 (1.8) ^1^	72 (1.2) ^1^	87 (1.1)	89 (1.2)
Per capita mean intake from snacks, mg (snackers only)	412 (15.5) ^1^	475 (35.8)	584 (36.9)	517 (24.1)	500 (25.4)	344 (9.3) ^1^	387 (20.5) ^1^	486 (21.4)	480 (20.7)	443 (22.1)
**>350% Federal Poverty Level**										
Snacks, *n*/day	1.2 (0.04) ^1^	1.4 (0.11) ^1^	2.2 (0.05)	2.7 (0.07)	2.5 (0.07)	1.2 (0.04) ^1^	1.4 (0.05) ^1^	1.7 (0.04) ^1^	2.4 (0.06)	2.6 (0.06)
Per capita mean intake from snacks, mg	278 (14.6) ^1^	297 (26.9) ^1^	497 (23.2)	556 (25.7) ^1^	427 (25.2)	215 (7.6) ^1^	251 (13.7) ^1^	354 (13) ^2^	435 (18.7)	411 (17.7)
% snackers	69 (1.3) ^1^	72 (3) ^1^	86 (1.3) ^1^	94 (0.9)	94 (0.8)	62 (1.2) ^1^	67 (1.7) ^1^	76 (1) ^1^	90 (0.8)	91 (0.8)
Per capita mean intake from snacks, mg (snackers only)	405 (19.2)	413 (37.6)	574 (24.9) ^1^	592 (28.1) ^1^	452 (27.2)	345 (9.7) ^1^	372 (19.3) ^1^	465 (13.7)	481 (19.2)	453 (18.7)
**Less than High School**										
Snacks, *n*/day	0.8 (0.04) ^1^	1.0 (0.05) ^1^	1.5 (0.09)	2.5 (0.1)	2.4 (0.08)	0.8 (0.03) ^1^	1.0 (0.04) ^1^	1.1 (0.04) ^1^	2.0 (0.07)	2.1 (0.07)
Per capita mean intake from snacks, mg	191 (13.6) ^1^	284 (29) ^1^	408 (28.5)	546 (23.4) ^2^	464 (28.6)	142 (7.8) ^1^	179 (16.3) ^1^	278 (14.1) ^1^	436 (28)	409 (21.6)
% snackers	48 (2.2) ^1^	56 (3.1) ^1^	73 (2.4) ^1^	89 (1)	88 (1.2)	47 (1.5) ^1^	55 (1.8) ^1^	64 (1.4) ^1^	84 (0.9)	84 (1.6)
Per capita mean intake from snacks, mg (snackers only)	397 (23.6)	504 (36.9)	556 (37.7)	611 (24.6) ^2^	528 (31.7)	303 (13.6) ^1^	322 (24.5) ^1^	437 (18.6)	517 (32.4)	486 (23.2)
**High School Diploma**										
Snacks, *n*/day	1.0 (0.03) ^1^	1.1 (0.07) ^1^	1.7 (0.06)	2.6 (0.08)	2.2 (0.07)	1.0 (0.03) ^1^	1.2 (0.07) ^1^	1.4 (0.05) ^1^	2.2 (0.06)	2.1 (0.07)
Per capita mean intake from snacks, mg	248 (10.5) ^1^	267 (27.8) ^1^	485 (33.6)	540 (27.7) ^1^	434 (20.4)	196 (8.5) ^1^	240 (16.5) ^1^	347 (17.7) ^1^	486 (34.4)	431 (25.5)
% snackers	61 (1.5) ^1^	61 (3) ^1^	79 (1.9) ^1^	90 (1.3)	88 (1.2)	57 (1.2) ^1^	61 (2) ^1^	70 (1.4) ^1^	88 (0.9)	86 (1.4)
Per capita mean intake from snacks, mg (snackers only)	407 (16.3)	439 (36.3)	614 (33.5) ^1^	598 (27.1) ^1^	492 (21.4)	345 (11.7) ^1^	392 (22) ^1^	494 (20.9)	549 (36.3)	503 (26.4)
**More than High School**										
Snacks, *n*/day	1.3 (0.04) ^1^	1.2 (0.1) ^1^	1.9 (0.05)	2.6 (0.05)	2.4 (0.06)	1.2 (0) ^1^	1.3 (0.1) ^1^	1.7 (0) ^1^	2.2 (0.1)	2.1 (0.1)
Per capita mean intake from snacks, mg	266 (11.3) ^1^	314 (24.4) ^1^	498 (24.9)	506 (13.3) ^1^	448 (15.7)	220.8 (7) ^1^	240 (12) ^1^	367 (12.8) ^1^	439 (13)	424 (13.3)
% snackers	70 (1.7) ^1^	68 (2.6) ^1^	83 (1.4) ^1^	90 (0.6)	89 (1.4)	63.2 (1.3) ^1^	65.6 (1.5) ^1^	75.8 (1.2) ^1^	89.9 (0.7)	90.5 (0.6)
Per capita mean intake from snacks, mg (snackers only)	390 (13.6) ^1^	463 (30.8) ^1^	594 (23.2) ^1^	548 (14.3) ^2^	491 (17.1)	349.3 (8.3) ^1^	366 (16.3) ^1^	484 (14.2)	488 (13.8)	468 (14)
**Hispanic**										
Snacks, *n*/day	0.9 (0.07) ^1^	1.6 (0.18) ^1^	2.1 (0.06)	2.6 (0.06)	2.4 (0.05)	0.9 (0.06) ^1^	0.9 (0.13) ^1^	1.2 (0.05) ^1^	2.0 (0.06) ^1^	2.3 (0.06)
Per capita mean intake from snacks, mg	187 (18.4) ^1^	334 (98.3) ^1^	419 (26.5)	534 (27.7)	469 (19.3)	153 (16.4) ^1^	222 (63.3) ^1^	286 (19.2) ^1^	386 (25.1)	403 (12.1)
% snackers	57 (3.1) ^1^	75 (6.4) ^1^	79 (1.8)	90 (0.9)	88 (1)	52 (2.7) ^1^	47 (5.5) ^1^	67 (1.9) ^1^	83 (1.3) ^1^	89 (0.9)
Per capita mean intake from snacks, mg (snackers only)	331 (26.4) ^1^	447 (131.2) ^1^	529 (29)	590 (28.1)	530 (19.5)	292 (28.2) ^1^	472 (122.4) ^1^	424 (26.8)	462 (27.6)	455 (15.8)
**Non-Hispanic White**										
Snacks, *n*/day	1.2 (0.03) ^1^	1.3 (0.06) ^1^	2.2 (0.04)	2.6 (0.06)	2.5 (0.06)	1.1 (0.03) ^1^	1.3 (0.06) ^1^	1.6 (0.04) ^1^	2.4 (0.06)	2.4 (0.06)
Per capita mean intake from snacks, mg	270 (8.7) ^1^	301 (19.8) ^1^	487 (24.6)	513 (17.4) ^1^	425 (19.5)	202 (6) ^1^	239 (10.4) ^1^	358 (10.8) ^1^	453 (18.1)	419 (15.1)
% snackers	66 (1.2) ^1^	68 (2.2) ^1^	85 (1.2)	92 (0.8)	92 (0.7)	60 (1.2) ^1^	64 (1.7) ^1^	74 (1) ^1^	90 (0.6)	89 (0.8)
Per capita mean intake from snacks, mg (snackers only)	410 (10.5) ^2^	441 (23.7)	572 (23.3) ^1^	559 (17.2) ^1^	463 (20.6)	335 (6.8) ^1^	372 (11.5) ^1^	484 (11.7)	505 (18.7)	468 (15.3)
**Non-Hispanic Black**										
Snacks, *n*/day	0.6 (0.05) ^1^	1.1 (0.11) ^1^	1.6 (0.06)	2.3 (0.06)	2.1 (0.04)	0.5 (0.04)	1.0 (0.08) ^1^	1.1 (0.07) ^1^	2.0 (0.04)	2.2 (0.06)
Per capita mean intake from snacks, mg	131 (12.9) ^1^	260 (45.1) ^1^	411 (26.6)	552 (22.3)	498 (27)	152 (19) ^1^	199 (22) ^1^	311 (25.2) ^1^	541 (30.5)	528 (27.5)
% snackers	39 (2.9) ^1^	59 (3.8) ^1^	74 (2.2)	88 (1)	86 (0.8)	37 (2.2) ^1^	55 (2.8) ^1^	61 (2.3) ^1^	86 (0.9)	86 (0.7)
Per capita mean intake from snacks, mg (snackers only)	333 (30.7) ^1^	441 (66)	556 (30.8)	626 (23.6)	580 (29.5)	415 (44.1) ^1^	360 (41.6) ^1^	514 (32.4) ^2^	633 (31.8)	616 (31.4)

^1^ Different from 2011 to 2014, *p* < 0.01; ^2^ Different from 2011 to 2014, *p* < 0.05. Note: Data come from the 1977–1978 Nationwide Food Consumption Survey (NFCS); the 1989–1991 Continuing Survey of Food Intake by Individuals (CSFII), the 1994–1996 CSFII, the 1997–1998 CSFII, NHANES 2003–2004, NHANES 2005–2006, NHANES 2009–2010 and NHANES 2011–2014. Results have been weighted to be nationally representative. Results are presented as mean (SE).

## Supplementary Materials

The following are available online at www.mdpi.com/2072-6643/9/6/610/s1, Figure S1: Contribution of salty snacks to sodium intake from snacking by race-ethnic group in US adults; Figure S2: Contribution of salty snacks to sodium intake from snacking by race-ethnic group in US children; Table S1: Socio-demographic characteristics for US adults and children from the 1977–1978 Nationwide Food Consumption Survey (NFCS), the 1989–1991 Continuing Survey of Food Intake by Individuals (CSFII), the 1994–1996 CSFII, the 1997–1998 CSFII, NHANES 2003–2004, NHANES 2005–2006, NHANES 2009–10 and NHANES 2011–2014; Table S2: Number of snacks consumed per day, percentage of snackers in the population and sodium consumed per snacking occasion by US children and adults from the 1977–1978, 1989–1991, 1994–1998, 2003–2006 and 2011–2014 surveys by BMI; Table S3: Top 10 sources of sodium intake from snack foods in US adults overall and by race-ethnic group; Table S4: Top 10 sources of sodium intake from snack foods in US children overall and by race-ethnic group.
