# Supplementary material for: Emerging Disparities in Dietary Sodium Intake from Snacking in the US Population
Source: Nutrients. 2017 Jun 17;9(6):610. doi: 10.3390/nu9060610 (PMC5490589; doi:10.3390/nu9060610)
Supplement: Supplementary file 1 [file nutrients-09-00610-s001.pdf]

Supplementary Table 1: Sociodemographic characteristics for US adults and children from the 1977–1978 Nationwide Food Consumption Survey (NFCS); the 1989–1991 Continuing Survey of Food Intake by Individuals (CSFII), the 1994–1996 CSFII, the 1997-1998 CSFII, NHANES 2003–2004, NHANES 2005– 2006, NHANES 2009-10 and NHANES 2011-14.

	NFCS 1977-78		CSFII 1989-91		CSFII 1994-98		NHANES 2003-06		NHANES 2011-14	
	**Children**	**Adults**	**Children**	**Adults**	**Children**	**Adults**	**Children**	**Adults**	**Children**	**Adults**
Number of observations	14,363	29,065	5,863	11,877	11,643	10,019	9,893	10,577	8,290	11,641
% Male	50.3	46.4	51.2	43.7	50.3	51.2	50.2	48.1	50.9	48.5
Race-ethnic status:										
Hispanic (%)	8	5	15	9	17	9	36	24	32	21
Non-Hispanic white (%)	76	84	66	78	66	79	28	50	25	39
Non-Hispanic black (%)	15	11	20	13	15	11	31	22	27	23
Income as % poverty										
<185% federal poverty level (%)	39	29	67	52	47	35	55	40	61	48
185-350 % federal poverty level (%)	40	37	22	24	28	28	22	25	19	22
>350% federal poverty level (%)	21	34	11	24	25	37	24	26	20	30
Body mass index										
Underweight (%)	43	4	45	4	57	2	44	2	53	2
Normal weight (%)	46	57	40	48	33	46	37	31	32	30
Overweight (%)	7	29	9	33	7	36	11	34	9	32
Obese (%)	4	10	6	16	4	19	8	34	6	36
Education household head										
Household's education <= high school diploma (%)	23	24	26	31	13	21	33	29	25	23
Household's education = high school diploma (%)	38	34	35	37	29	35	25	25	23	22
Household’s education >= high school diploma (%)	40	41	39	32	58	44	42	46	52	55

Supplementary Table 2: **Number of snacks consumed per day, percentage snackers in the population and sodium consumed per snacking occasion by US children and adults from the 1977-78, 1989-91, 1994-98, 2003-06 and 2011-14 surveys by BMI**

	Children					Adults				
	1977-78	1989-91	1994-98	2003-06	2011-14	1977-78	1989-91	1994-98	2003-06	2011-14
**Underweight**										
Snacks, n/d	1.2 (.03)	1.4 (.08)	2.4 (.05)	2.8 (.07)	2.7 (.06)	1.1 (.07)	1.2 (.07)	1.3 (.11)	2.3 (.14)	2.4 (.15)
Total sodium from snacks, mg	209 (7.7)[1]	250 (19.6)[1]	425 (19.8)	529 (18)[1]	460 (16)	196 (23.6)[1]	208 (25.3)[1]	329 (57.3)[2]	638 (123.3)	539 (60)
% snackers	64 (1.3)	66 (2.4)	86 (1.2)	95 (.6)	95 (.8)	57 (2.5)	65 (2.8)	68 (3.6)	89 (2.7)	86 (3.7)
Sodium per snack, mg	324 (9.5)[1]	377 (25.2)[1]	492 (18.7)[1]	560 (18.6)[1]	486 (17.5)	345 (38.2)[1]	320 (37.3)[1]	482 (76)	718 (145.2)	625 (63.7)
**Normal weight**										
Snacks, n/d	1.0 (.03)	1.3 (.06)	1.9 (.05)	2.5 (.06)	2.3 (.06)	1.1 (.03)	1.3 (.05)	1.6 (.04)	2.4 (.05)	2.4 (.07)
Total sodium from snacks, mg	284 (11.7)[1]	333 (28)[1]	526 (28.8)	634 (28.3)[1]	489 (23.9)	202 (6.1)[1]	240 (11.5)[1]	363 (14.9)[1]	496 (29.1)	444 (21.2)
% snackers	60 (1.3)	66 (2.3)	82 (1.3)	91 (.8)	91 (.9)	59 (1.1)	62 (1.5)	74 (1.2)	89 (1)	90 (.9)
Sodium per snack, mg	475 (16.6)[2]	507 (37.6)	645 (29.1)	696 (30.5)[1]	538 (26.5)	339 (7.9)[1]	384 (15.2)[1]	493 (16.7)	555 (32.2)	495 (21.6)
**Overweight**										
Snacks, n/d	0.9 (.04)	1.1 (.1)	1.7 (.07)	2.2 (.08)	2.0 (.1)	1.0 (.03)	1.2 (.06)	1.5 (.04)	2.3 (.05)	2.4 (.05)
Total sodium from snacks, mg	223 (17)[1]	236 (31.7)[1]	500 (54.3)	500 (39.3)	471 (56.8)	186 (8.1)[1]	215 (12.9)[1]	337 (13.1)[1]	435 (17.8)	412 (16.8)
% snackers	54 (2.3)	60 (4)	73 (2.7)	89 (1.8)	85 (3)	55 (1.4)	61 (1.8)	71 (1.1)	88 (.7)	88 (1)
Sodium per snack, mg	415 (27.1)	395 (44.7)[2]	681 (67.2)	563 (41.5)	551 (63.3)	337 (10.4)[1]	350 (17.5)[1]	477 (17.1)	493 (18.6)	466 (18)
**Obese**										
Snacks, n/d	1.0 (.08)	1.2 (.12)	1.8 (.13)	2.0 (.07)	2.1 (.13)	0.9 (.04)	1.2 (.06)	1.4 (.05)	2.2 (.07)	2.3 (.06)
Total sodium from snacks, mg	207 (23.8)[1]	243 (50.8)[1]	368 (55.5)	411 (31.1)	463 (45.5)	173 (12.8)[1]	222 (16.1)[1]	350 (19.6)[1]	415 (19.6)	414 (13.4)
% snackers	56 (3.6)	63 (5.3)	73 (3.9)	90 (2.1)	87 (2.3)	52 (1.9)	60 (2.4)	71 (1.6)	88 (.9)	88 (1)

Sodium per snack, mg	372 (41.6)[2]	387 (75.1)	503 (65.8)	454 (32.4)	533 (56.2)	333 (20.9)[1]	368 (21.7)[1]	491 (23.1)	470 (21)	471 (15.5)

Supplementary Table 3: **Top 10 sources of sodium intake from snack foods in US adults overall and by race-ethnic group**

	1977-78	1989-91	1994-98	2003-06	2011-14
**Overall**					
1	Grain-based desserts	Salty snacks	Salty snacks	Salty snacks	Grain-based desserts
2	Salty snacks	Grain-based desserts	Grain-based desserts	Grain-based desserts	Salty snacks
3	Processed meats and poultry	Breads and rolls	Processed meats and poultry	Processed meats and poultry	Processed meats and poultry
4	Breads and rolls	Fluid milk	Breads and rolls	Breads and rolls	Tortilla and corn-based dishes
5	Cheese & cheese products	Cheese & cheese products	Cheese & cheese products	Cheese & cheese products	Cheese & cheese products
6	Fluid milk	Processed meats and poultry	Dairy-based desserts	Pizza & calzone	Breads and rolls
7	Dairy-based desserts	Dairy-based desserts	Fluid milk	Tortilla and corn-based dishes	Nuts, nut butters, seeds, & coconut
8	Soups & stews	Soups & stews	Pizza & calzone	Fluid milk	Dairy-based desserts
9	Nuts, nut butters, seeds, & coconut	Pizza & calzone	Soups & stews	Dairy-based desserts	Fluid milk
10	Coffee/tea	Sugar sweetened beverages	Nuts, nut butters, seeds, & coconut	Nuts, nut butters, seeds, & coconut	Sandwiches & filled rolls
**Hispanic**					
1	Grain-based desserts	Grain-based desserts	Grain-based desserts	Grain-based desserts	Grain-based desserts
2	Processed meats and poultry	Salty snacks	Salty snacks	Salty snacks	Tortilla and corn-based dishes
3	Breads and rolls	Fluid milk	Processed meats and poultry	Tortilla and corn-based dishes	Salty snacks
4	Fluid milk	Cheese & cheese products	Rice and other grains and dishes	Processed meats and poultry	Cheese & cheese products
5	Salty snacks	Processed meats and poultry	Fluid milk	Breads and rolls	Breads and rolls
6	Cheese & cheese products	Breads and rolls	Breads and rolls	Fluid milk	Processed meats and poultry

7	Rice and other grains and dishes	Soups & stews	Tortilla and corn-based dishes	Pizza & calzone	Fluid milk
8	Coffee/tea	Dips & spreads	Sandwiches & filled rolls	Soups & stews	Pizza & calzone
9	Dairy-based desserts	Coffee/tea	Soups & stews	Cheese & cheese products	Soups & stews
10	Nuts, nut butters, seeds, & coconut	Tortilla and corn-based dishes	Meat	Nuts, nut butters, seeds, & coconut	Dairy-based desserts
**Non-Hispanic White**					
1	Grain-based desserts	Salty snacks	Salty snacks	Salty snacks	Grain-based desserts
2	Salty snacks	Grain-based desserts	Grain-based desserts	Grain-based desserts	Salty snacks
3	Processed meats and poultry	Fluid milk	Processed meats and poultry	Cheese & cheese products	Cheese & cheese products
4	Breads and rolls	Breads and rolls	Breads and rolls	Processed meats and poultry	Processed meats and poultry
5	Cheese & cheese products	Cheese & cheese products	Cheese & cheese products	Breads and rolls	Breads and rolls
6	Fluid milk	Dairy-based desserts	Dairy-based desserts	Dairy-based desserts	Nuts, nut butters, seeds, & coconut
7	Dairy-based desserts	Soups & stews	Fluid milk	Pizza & calzone	Fluid milk
8	Nuts, nut butters, seeds, & coconut	Processed meats and poultry	Nuts, nut butters, seeds, & coconut	Fluid milk	Dairy-based desserts
9	Soups & stews	Pizza & calzone	Soups & stews	Tortilla and corn-based dishes	Tortilla and corn-based dishes
10	Coffee/tea	Nuts, nut butters, seeds, & coconut	Sugar sweetened beverages	Nuts, nut butters, seeds, & coconut	Pizza & calzone
**Non-Hispanic Black**					
1	Processed meats and poultry	Salty snacks	Grain-based desserts	Salty snacks	Grain-based desserts
2	Grain-based desserts	Grain-based desserts	Salty snacks	Grain-based desserts	Salty snacks
3	Breads and rolls	Processed meats and poultry	Processed meats and poultry	Processed meats and poultry	Processed meats and poultry

4	Sandwiches & filled rolls	Breads and rolls	Pizza & calzone	Breads and rolls	Breads and rolls
5	Meat	Meat	Breads and rolls	Pizza & calzone	Sandwiches & filled rolls
6	Salty snacks	Dairy-based desserts	Poultry	Cheese & cheese products	Cheese & cheese products
7	Cheese & cheese products	Soups & stews	Meat	Poultry	Poultry
8	Fluid milk	Fluid milk	Sugar sweetened beverages	Tortilla and corn-based dishes	Tortilla and corn-based dishes
9	Soups & stews	Sugar sweetened beverages	Dairy-based desserts	Sandwiches & filled rolls	Pizza & calzone
10	Dairy-based desserts	Poultry	Cheese & cheese products	Meat	Dairy-based desserts

<u>Supplementary Table 4:</u> Top 10 sources of sodium intake from snack foods in US children overall and by race-ethnic group

	1977-78	1989-91	1994-98	2003-06	2011-14
**Overall**					
1	Grain-based desserts	Salty snacks	Salty snacks	Salty snacks	Salty snacks
2	Fluid milk	Grain-based desserts	Grain-based desserts	Grain-based desserts	Grain-based desserts
3	Salty snacks	Fluid milk	Fluid milk	Processed meats and poultry	Fluid milk
4	Breads and rolls	Processed meats and poultry	Processed meats and poultry	Fluid milk	Tortilla and corn-based dishes
5	Processed meats and poultry	Breads and rolls	Cheese & cheese products	Tortilla and corn-based dishes	Processed meats and poultry
6	Dairy-based desserts	Cheese & cheese products	Baby food, infant food, formula	Breads and rolls	Cheese & cheese products
7	Cheese & cheese products	Soups & stews	Breads and rolls	Cheese & cheese products	Breads and rolls
8	Pizza & calzone	Ready-to-eat cereals	Ready-to-eat cereals	Pizza & calzone	Pizza & calzone
9	Soups & stews	Dairy-based desserts	Dairy-based desserts	Soups & stews	Sandwiches & filled rolls
10	Ready-to-eat cereals	Pizza & calzone	Soups & stews	Dairy-based desserts	Dairy-based desserts
**Hispanic**					
1	Fluid milk	Fluid milk	Fluid milk	Salty snacks	Salty snacks
2	Grain-based desserts	Soups & stews	Salty snacks	Grain-based desserts	Grain-based desserts
3	Breads and rolls	Grain-based desserts	Grain-based desserts	Tortilla and corn-based dishes	Fluid milk

4	Ready-to-eat cereals	Salty snacks	Baby food, infant food, formula	Fluid milk	Tortilla and corn-based dishes
5	Processed meats and poultry	Ready-to-eat cereals	Ready-to-eat cereals	Processed meats and poultry	Processed meats and poultry
6	Soups & stews	Processed meats and poultry	Cheese & cheese products	Breads and rolls	Cheese & cheese products
7	Salty snacks	Cheese & cheese products	Processed meats and poultry	Soups & stews	Breads and rolls
8	Cheese & cheese products	Breads and rolls	Soups & stews	Cheese & cheese products	Pizza & calzone
9	Dairy-based desserts	Tortilla and corn-based dishes	Tortilla and corn-based dishes	Pizza & calzone	Soups & stews
10	Nuts, nut butters, seeds, & coconut	Baby food, infant food, formula	Breads and rolls	Ready-to-eat cereals	Ready-to-eat cereals
**Non-Hispanic White**					
1	Grain-based desserts	Salty snacks	Salty snacks	Salty snacks	Salty snacks
2	Salty snacks	Grain-based desserts	Grain-based desserts	Grain-based desserts	Grain-based desserts
3	Fluid milk	Fluid milk	Fluid milk	Fluid milk	Fluid milk
4	Processed meats and poultry	Processed meats and poultry	Cheese & cheese products	Cheese & cheese products	Processed meats and poultry
5	Breads and rolls	Cheese & cheese products	Processed meats and poultry	Processed meats and poultry	Cheese & cheese products
6	Dairy-based desserts	Breads and rolls	Breads and rolls	Breads and rolls	Tortilla and corn-based dishes

7	Pizza & calzone	Soups & stews	Baby food, infant food, formula	Pizza & calzone	Breads and rolls
8	Cheese & cheese products	Dairy-based desserts	Dairy-based desserts	Dairy-based desserts	Dairy-based desserts
9	Soups & stews	Pizza & calzone	Ready-to-eat cereals	Tortilla and corn-based dishes	Candy
10	Ready-to-eat cereals	Ready-to-eat cereals	Pizza & calzone	Candy	Sandwiches & filled rolls
**Non-Hispanic Black**					
1	Grain-based desserts	Salty snacks	Salty snacks	Salty snacks	Salty snacks
2	Fluid milk	Grain-based desserts	Grain-based desserts	Grain-based desserts	Grain-based desserts
3	Salty snacks	Fluid milk	Processed meats and poultry	Processed meats and poultry	Processed meats and poultry
4	Breads and rolls	Breads and rolls	Fluid milk	Pizza & calzone	Tortilla and corn-based dishes
5	Processed meats and poultry	Processed meats and poultry	Breads and rolls	Breads and rolls	Pizza & calzone
6	Sandwiches & filled rolls	Cheese & cheese products	Cheese & cheese products	Cheese & cheese products	Sandwiches & filled rolls
7	Dairy-based desserts	Pasta, noodle dishes	Baby food, infant food, formula	Tortilla and corn-based dishes	Breads and rolls
8	Nuts, nut butters, seeds, & coconut	Pizza & calzone	Soups & stews	Fluid milk	Cheese & cheese products
9	Ready-to-eat cereals	Soups & stews	Sandwiches & filled rolls	Soups & stews	Poultry

10	Sugar sweetened beverages	Baby food, infant food, formula	Dairy-based desserts	Sugar sweetened beverages	Fluid milk

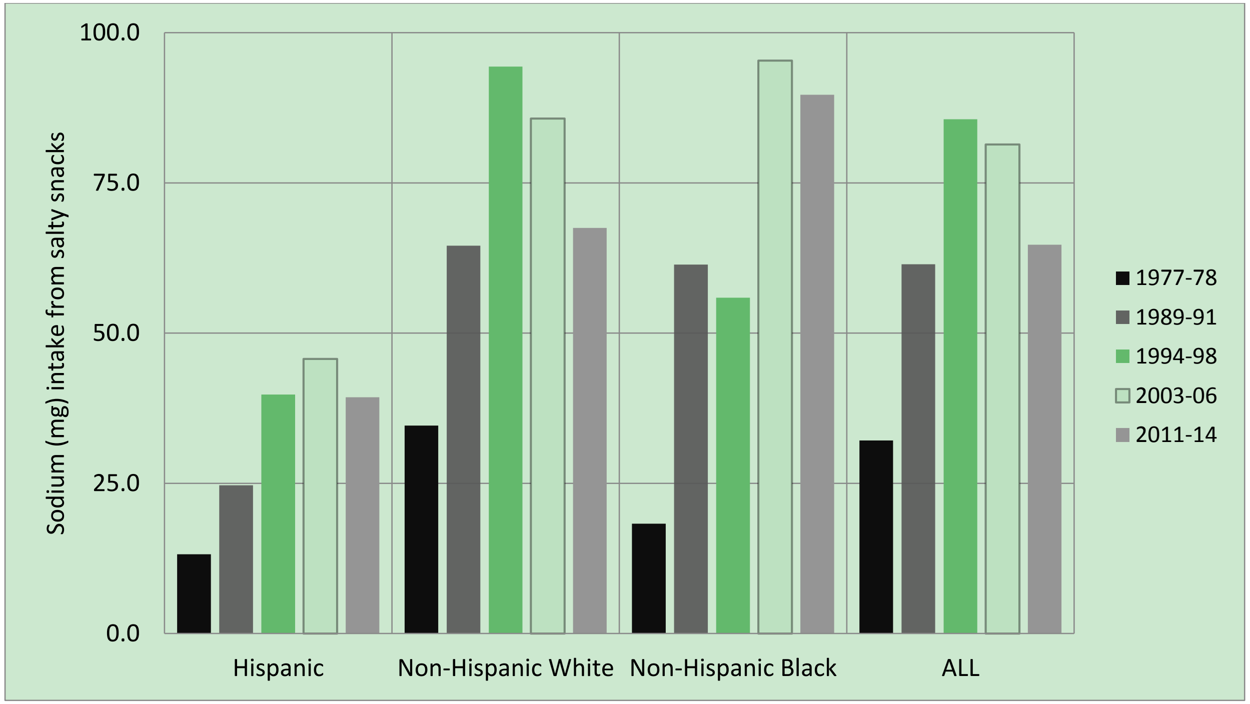

<u>Supplementary Figure 1:</u> Contribution of salty snacks to sodium intake from snacking by race-ethnic group in US adults

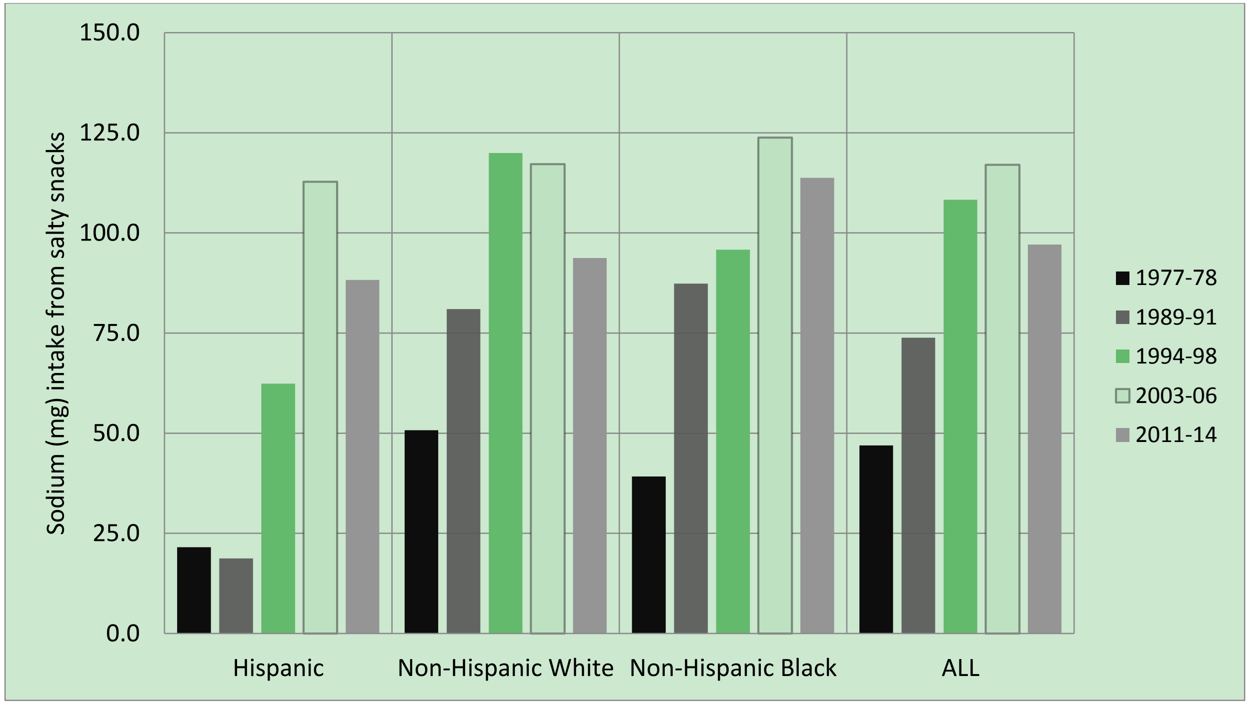

<u>Supplementary Figure 2:</u> Contribution of salty snacks to sodium intake from snacking by race-ethnic group in US children
